# Monitoring of Host Suitability and Defense-Related Genes in Wheat to *Bipolaris sorokiniana*

**DOI:** 10.3390/jof8020149

**Published:** 2022-01-31

**Authors:** Mehtap Alkan, Harun Bayraktar, Mustafa İmren, Fatih Özdemir, Rachid Lahlali, Fouad Mokrini, Timothy Paulitz, Abdelfattah A. Dababat, Göksel Özer

**Affiliations:** 1 Department of Plant Protection, Faculty of Agriculture, Bolu Abant Izzet Baysal University, Bolu 14030, Turkey; alkanmhtp@gmail.com (M.A.); m.imren37@gmail.com (M.İ.); 2 Department of Plant Protection, Faculty of Agriculture, Ankara University, Ankara 06110, Turkey; 3 Bahri Dagdas International Agricultural Research Institute, Konya 42050, Turkey; ozdemirfatih@tarimorman.gov.tr; 4 Phytopathology Unit, Department of Plant Protection, Ecole Nationale d’Agriculture de Meknès, BPS 40, Meknes 50001, Morocco; rlahlali@enameknes.ac.ma; 5 Biotechnology Research Unit, Laboratory of Nematology, Regional Center of Agricultural Research, National Institute of Agronomic Research (INRA), Rabat 10060, Morocco; fmokrini.inra@gmail.com; 6 Wheat Health, Genetics and Quality Research Unit, United States Department of Agriculture, Agricultural Research Service, Washington State University, Pullman, WA 99164, USA; timothy.paulitz@usda.gov; 7 International Maize and Wheat Improvement Centre (CIMMYT), P.O. Box 39, Emek, Ankara 06170, Turkey

**Keywords:** disease reaction, gene expression, pathogen quantification, qPCR, wheat, spot blotch disease

## Abstract

Spot blotch caused by *Bipolaris sorokiniana* is a destructive disease of wheat worldwide. This study investigated the aggressiveness of *B. sorokiniana* isolates from different wheat-growing areas of Bolu province in Turkey on the cultivar Seri-82. Host susceptibility of 55 wheat cultivars was evaluated against the most aggressive isolate. Our results indicated that the cultivars Anafarta and Koç-2015 were the most resistant. A specific and sensitive qPCR assay was developed for detecting the pathogen in plant tissues and evaluating wheat plants with different resistance levels. Three primer sets, BsGAPDHF/BsGAPDHR, BsITSF/BsITSR, and BsSSUF/BsSSUR, were designed based on glyceraldehyde-3-phosphate dehydrogenase, internal transcribed spacers, and 18S rRNA loci of *B. sorokiniana* with detection limits of 1, 0.1, and 0.1 pg of pathogen DNA, respectively. The qPCR assay was highly sensitive and did not amplify DNA from the other closely related fungal species and host plants. The protocol differentiated wheat plants with varying degrees of resistance. The assay developed a useful tool for the quantification of the pathogen in the early stages of infection and may provide a significant contribution to a more efficient selection of wheat genotypes in breeding studies. In the present study, expression levels of PR proteins, phenylalanine ammonia-lyase, catalase, ascorbate peroxidase, and superoxide dismutase enzymes were upregulated in Anafarta (resistant) and Nenehatun (susceptible) cultivars at different post-infection time points, but more induced in the susceptible cultivar. The results showed considerable variation in the expression levels and timing of defense genes in both cultivars.

## 1. Introduction

Wheat (*Triticum* spp.) is among the most important food sources for human food and the most widely grown crop in the world. Many biotic factors continuously challenge wheat production worldwide [1,2]. Among the fungal diseases, spot blotch and common root rot caused by *Bipolaris sorokiniana* (Sacc. in Sorokin) Shoemaker (teleomorph: *Cochliobolus sativus* (S. Ito and Kuribayashi) Drechsler ex Dastur) is a significant disease that occurs widely in wheat-growing areas worldwide and affects leaf, head, stem, and root tissue of wheat [3]. The pathogen survives from season to season in infected seed, soil, or plant debris and causes economically important losses of up to 100% in yield and quality of wheat under favorable conditions [2,3]. In Turkey, the disease occurs widely and causes serious damage in wheat-growing areas as well as in the rest of the world [2,4,5].

The use of resistant varieties is considered the most beneficial and eco-friendly for managing spot blotch disease [6]. However, genetic and pathogenic variation within pathogen populations complicated the studies of resistance breeding [7,8]. Thus, more detailed studies are needed to increase control methods’ effectiveness and better understand the host–pathogen relationship. Assessment of host response to different pathogen populations is indispensable to reveal plant–pathogen interaction. Several studies have been performed to estimate the disease severity of *B. sorokiniana* by using different disease assessment methods [9,10,11] to reveal resistance sources. These screening methods are affected by environmental conditions plants growth stage and assessing experts, which cause difficulties in the appropriate assessment of host resistance. Therefore, more reliable and repeatable screening techniques are needed to evaluate disease reactions in plants. Additionally, fast and reliable identification of fungi responsible for plant diseases at an early stage of disease development is important to improve disease management practices. Many techniques have been used routinely for disease detection and pathogen identification [12,13]. Molecular techniques have become a useful approach to study the phylogenetic relationship among plant pathogens and conserved sequences of internal transcribed spacers (ITS), *β-tubulin*, 28S rRNA gene (LSU), 18S rRNA gene (SSU), RNA polymerase second largest subunit (*RPB2*), translation elongation factor 1-alpha (*EF1-α*), and glyceraldehyde-3-phosphate dehydrogenase (*GAPDH*) provided informative sites for the specific detection of numerous fungal diseases [14,15,16]. Different approaches based on PCR were used for routine detection of spot blotch disease [17,18,19,20]. However, the development of more novel disease recognition and assessment techniques is needed to improve the efficiency of plant disease management practices.

Plants have developed sophisticated mechanisms to recognize attack by pathogens and activate an effective natural immune response. The understanding of signal transduction genes that play a key role in the resistance mechanisms of plants is essential for the development of novel disease management strategies associated with host resistance. Plant–pathogen interaction is known to induce defense responses including the generation of reactive oxygen species, phytoalexin biosynthesis, cell wall cross-linking, synthesis of defense enzymes, and the accumulation of pathogenesis-related (PR) proteins [21,22,23]. PR proteins involved in defense mechanism during plant–pathogen interaction have been widely recognized and clustered into 17 groups linked to amino acid sequences, serological characteristics, and enzymatic activities [24,25,26]. Thus far, many studies have been carried out to reveal the relationship between PR proteins and stress defense mechanisms of plants on the basis of gene expression [26,27,28,29]. The overexpression of *PR* genes in wheat plants resulted in increased resistance to several pathogens, i.e., *Fusarium graminearum* (FHB), *Puccinia* spp., and *Blumeria graminis* [30,31,32,33]. Similarly, the role of phenylalanine ammonia-lyase (*PAL*) involving in the production of defense-related compounds and guaiacol peroxidase (*POD*), catalase (*CAT*), ascorbate peroxidase (*APX*), and superoxide dismutase (*SOD*) enzymes associated with reactive oxygen species (ROS) detoxification has been indicated in a number of plant–pathogen interactions [34,35,36].

Therefore, this study aimed to: (i) obtain and characterize *B. sorokiniana* isolates from wheat fields in Bolu province, (ii) develop a qPCR assay for fast and reliable detection of pathogen infection in plant tissues at the early stages and monitoring of wheat genotypes with different resistance levels, to reveal disease reaction of common Turkish wheat cultivars as candidate plants for breeding studies, and (iii) evaluate the transcription level of several PR proteins (*PR1*, *PR2*, *PR3*, *PR5*, and *PR10*) and defense enzymes (*PAL*, *CAT*, *SOD*, and *APX*) that play a role in defense response of wheat plants with different resistance levels to spot blotch disease caused by *B. sorokiniana*.

## 2. Materials and Methods

### 2.1. Fungal Material

The fungal isolates were recovered from diseased leaf and root samples collected from different wheat fields located in Bolu province, Turkey. Symptomatic tissues showing leaf spot, root, and crown rot were surface sterilized in a 1% sodium hypochlorite solution for 2 min, rinsed twice with sterile distilled water, and placed in Petri dishes containing 1/5 strength potato dextrose agar (PDA) medium amended with 100 mg/L streptomycin sulphate and 25 mg/L chloramphenicol to inhibit bacterial growth. The Petri dishes were incubated for 3 days at 23 ± 1 °C in the dark. The growing cultures were examined on a light microscope (DM1000, Leica Microsystems, Wetzlar, Germany) and transferred to a new PDA medium. The isolates derived from hyphal tips were preserved on filter paper at 4 °C. Morphological identification of the fungal isolates was performed according to the criteria of Sivanesen [37]. The other fungal pathogens associated with wheat plant in previous studies, *Fusarium culmorum* (Wm.G. Sm.) Sacc., *Fusarium graminearum* Schwabe, *Fusarium pseudograminearum* Aoki and O’Donnell, *Fusarium oxysporum* Schltdl, *Fusarium acuminatum* Ellis ve Everh., *Fusarium equiseti* (Corda) Sacc., *Fusarium sambucinum* Fuckel, *Fusarium avenaceum* (Fr.) Sacc., *Alternaria alternata* (Fr.) Keissl., *Pyrenophora teres* Drechsler, *Pyrenophora tritici-repentis* (Died.) Drechsler, *ZymoSeptoria tritici* (Desm.) Quaedvlieg and Crous, *Macrophomina phaseolina* (Tassi) Goid, and *Rhizoctonia solani* J.G. Kühn, were included in the study to control the specificity of the designed primers.

### 2.2. DNA Extraction

DNA isolation from approximately 100 mg of frozen leaf tissue or mycelial mat was performed using DNeasy Plant Mini Kit (Qiagen, Hilden, Germany) or DNeasy Blood & Tissue Kit (Qiagen, Hilden, Germany) according to the manufacturer’s protocol. The concentration and quality of DNA was measured using a DS-11 FX+ spectrophotometer (Denovix Inc., Wilmington, DE, USA) and diluted to 10 ng/μL with ultrapure ddH_2_O. To quantify the amount of pathogen, a standard calibration curve was obtained using a 10-fold serial dilution of fungal DNA with healthy plant DNA. DNA concentrations in the calibration curve ranged from 10 ng to 0.01 pg/μL and were used in qPCR reactions as an internal control of DNA quantification.

### 2.3. DNA Sequencing and Phylogeny

Morphological identification of the pathogen isolates was also confirmed by DNA sequencing of the rDNA ITS and *GAPDH* loci with the primer pairs ITS1/ITS4 [38] and gpd1/gpd2 [39], respectively. Amplification was performed in a total volume of 50 μL containing 1× PCR buffer, 2 mM MgCl_2_, 0.4 μM each primer, 0.2 mM dNTPs, and 1.25 U Taq DNA polymerase (New England Biolabs, Beverly, MA, USA). The PCR amplifications consisted of an initial denaturation of one cycle for 3 min at 95 °C, followed by 35 cycles of 1 min at 95 °C, 1 min at 52 °C, and 1 min at 72 °C, while the final extension step was carried out at 72 °C for 10 min. PCR products were visualized on 1.4% agarose gel using 1× TAE buffer and sequenced in both directions using the same primers by Macrogen company (Seoul, Korea). The sequences obtained were examined by BLAST analysis using the NCBI website (http://blast.ncbi.nlm.nih.gov/Blast.cgi, accessed on 10 December 2021). Sequences were read and edited with MegAlign module of DNASTAR software version 7.1.0 (DNASTAR Inc., Madison, WI, USA), and deposited in the NCBI GenBank nucleotide database (https://www.ncbi.nlm.nih.gov/genbank/, accessed on 10 December 2021).

For phylogenetic reconstruction, the sequences of *ITS* and *GAPDH* were compared with published *Bipolaris* and *Curvularia* spp. sequences. Preliminary sequence alignments of two individual loci (*ITS* and *GAPDH*) were generated using MAFFT v. 7.490 [40] (http://mafft.cbrc.jp/alignment/server/index.html, accessed on 10 December 2021) with the default parameters. Phylogenetic analysis based on the combined alignment of the two loci of the isolates from the current study and those of reference isolates from GenBank were reconstructed using Maximum-Likelihood (ML) analysis with MEGA 7 [41]. The bootstrap re-sampling analysis for 1000 replicates was used to estimate the confidence of tree topologies [42]. Phylogenetic trees were rooted to *Pyrenophora tritici-repentis* (ITS: AF071348; *GAPDH*: AF081370) and visualized with MEGA 7.

### 2.4. Pathogenicity Tests of Isolates and Host Susceptabilty of Wheat Cultivars 

All isolates from different fields were subjected to a preliminary pathogenicity test on the susceptible wheat cultivar Seri-82. To prepare the inoculum, the isolates were grown on PDA medium in the dark at 23 °C for 7 days. Mycelial plugs from the edge of the cultures were transferred into the polyester bags containing 200 g of sterilized wheat seeds and incubated at 23 °C for 15 days in the dark. To ensure homogeneity, the bags were turned upside down every two days. The inoculum was harvested by mixing the inoculated wheat seeds with 200 mL of distilled water on a magnetic shaker and filtering through sterile filter paper. Spore concentration was adjusted to a final concentration of 1 × 10^4^ conidia/mL by diluting in sterile distilled water.

Wheat seeds were surface sterilized with a 1% sodium hypochlorite solution for 2 min, rinsed with distilled water and sown in pots (17 cm in length and 11 cm in diameter) containing sterilized compost. Three pots for each isolate were grown at 23 °C under a 12-h photoperiod. Fifteen days after sowing, plants were sprayed with spore suspensions until run-off. Plants were immediately covered with a transparent plastic bag to maintain humidity and maintained at 20 °C for 18 h. The inoculated plants were incubated at 23 °C for 12 days. Disease severity of wheat plants was evaluated using a 1–9 scale as per Fetch and Steffenson [11]. The infection response was classified into three general categories of low, intermediate, and high host-parasite compatibility. The infection response 1, 2 and 3 were considered indicative of low compatibility; 4 and 5 were intermediate compatibility; and 6, 7, 8, and 9 were high compatibility. The data obtained from disease scores were also analyzed using Statistical Analysis System computer software (SAS Version 9.0; SAS Institute Inc.; Cary, NC, USA). Means values were separated according to Tukey’s HSD method (Honestly Significant Difference (HSD) test.

Using the most aggressive isolates in a population derived from specific regions or provinces is desirable to ensure the most effective line screening in wheat and barley breeding programs. The most aggressive isolate was used to evaluate the disease reaction of 55 wheat cultivars that are widely cultivated in Turkey (Table 1). The disease severity data were subjected to analysis with Levene’s homogeneity of variance test and then one-way ANOVA, followed by Tukey HSD test (*p* ≤ 0.05) contained in the SAS software.

### 2.5. Primer Design 

The reference sequences of the ITS, *GAPDH*, and SSU region of *B. sorokiniana* and closely related fungi were retrieved from GenBank and aligned for the conserved region together with the obtained sequences in this study by using MEGA 7 software. Species-specific primer sets were designed by using Primer3 ([43], https://bioinfo.ut.ee/primer3-0.4.0/, accessed on 10 December 2021) and UNAFold software (https://eu.idtdna.com/unafold/, accessed on 10 December 2021). The specificity of the primers was also confirmed by NCBI-BLAST analysis for searching the primer sequences.

### 2.6. Detection of Bipolaris sorokiniana Infection in Wheat Tissues

The reaction of the resistant (Altay, Koç-2015, and Anafarta) and susceptible (Kırik, Damla, and Nenehatun) wheat cultivars to spot blotch disease was also evaluated by qPCR assay with the designed primer sets. The inoculation was performed as mentioned above. The second leaves of wheat plants from each pot were collected at 3, 5, and 8 days after inoculation, washed with sterile water, placed into 50 mL falcon tubes, and immediately frozen in liquid nitrogen. DNA extraction was carried out as described above.

Real-time PCR assay was performed using a BioRad CFX96 Real-time PCR system (Bio-Rad Laboratories Inc., Hercules, CA, USA). PCR mixtures consisted of 2 μL diluted cDNA, 0.6 μM primer, 1 × iTaq Universal SYBR Green Supermix (Bio-Rad Laboratories Inc., Hercules, CA, USA) in 20 μL volume. The PCR amplifications consisted of an initial denaturation of one cycle for 3 min at 95 °C, 45 cycles of 10 s at 95 °C, and 30 s at 60 °C, followed by denaturation for melting curve analysis. The amount of pathogen DNA in plant tissues was quantified using the standard curves constructed with known concentrations of pathogen DNA, from 10 ng to 0.01 pg.

### 2.7. Expression Analysis of Defense-Related Genes in Wheat to the Pathogen

The transcript level of defense genes was evaluated in Anafarta (resistant) and Nenehatun (susceptible) wheat cultivars. The second leaves of inoculated plants were collected at 12, 24, 48, 72, and 96 h post-inoculation (hpi). The samples were immediately frozen in liquid nitrogen and stored until used for RNA isolation. Mock inoculations were performed only with distilled water. Three pots were used for each inoculation point and leaves from 10 plants for each replicate were pooled.

RNA extraction was performed from 100 mg of leave tissue using NucleoZOL (Macherey-Nagel, Düren, Germany) RNA isolation buffer following the manufacturer’s specifications. To eliminate residual DNA, the samples were treated with 1 U of DNase I (Thermo Fisher Scientific, Waltham, MA, USA) according to the protocol recommended by the manufacturer. RNA quality was evaluated using the DS-11 FX+ spectrophotometer. After the purification process, cDNA synthesis was performed from the mRNA using an iScript™ cDNA synthesis kit (Bio-Rad Laboratories Inc., Hercules, CA, USA) according to the manufacturer’s instructions. For gene expression analysis with qRT-PCR, all cDNA samples were diluted 10-fold with sterile ultrapure water.

The qRT-PCR reaction comprised of 2 μL cDNA, 0.5 μM primer, 1 × Sso Advanced SYBR Green Supermix (Bio-Rad Laboratories Inc., Hercules, CA, USA) in 10 μL volume. The cycling condition was 3 min at 95 °C, 45 cycles of 10 s at 95 °C, and 10 s at 60 °C. After the ending of the PCR assay, a denaturation curve from 65 to 95 °C was performed. The housekeeping gene *β-tubulin* was used as an internal control for relative quantification in all analyses (Table 2). Three biological replicates and two technical replicates were analyzed for each gene. The expression level of the gene was determined according to the 2 ^−ΔΔCT^ method of Livak and Schmittgen [44] using Ct-values of *β-tubulin* gene for normalization. The fold changes were subjected to ANOVA analysis or Student’s *t*-test analysis using Minitab 17 statistical software for Windows (Minitab, Inc.: State College, PA, USA, www.minitab.com, accessed on 2 January 2022).

## 3. Results

### 3.1. The Identification of Pathogen Isolates

**Figure 1:**
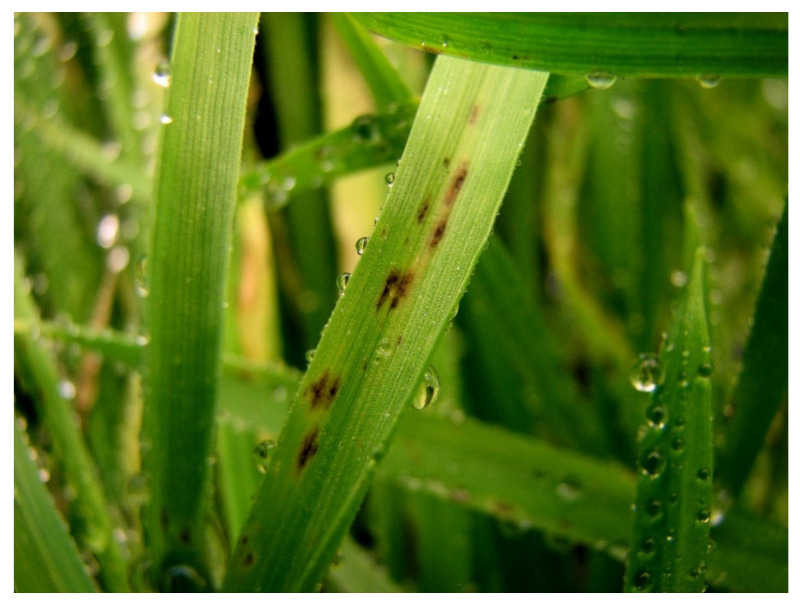
Typical spot blotch symptom caused by *Bipolaris sorokiniana* on wheat plants.

**Figure 2:**
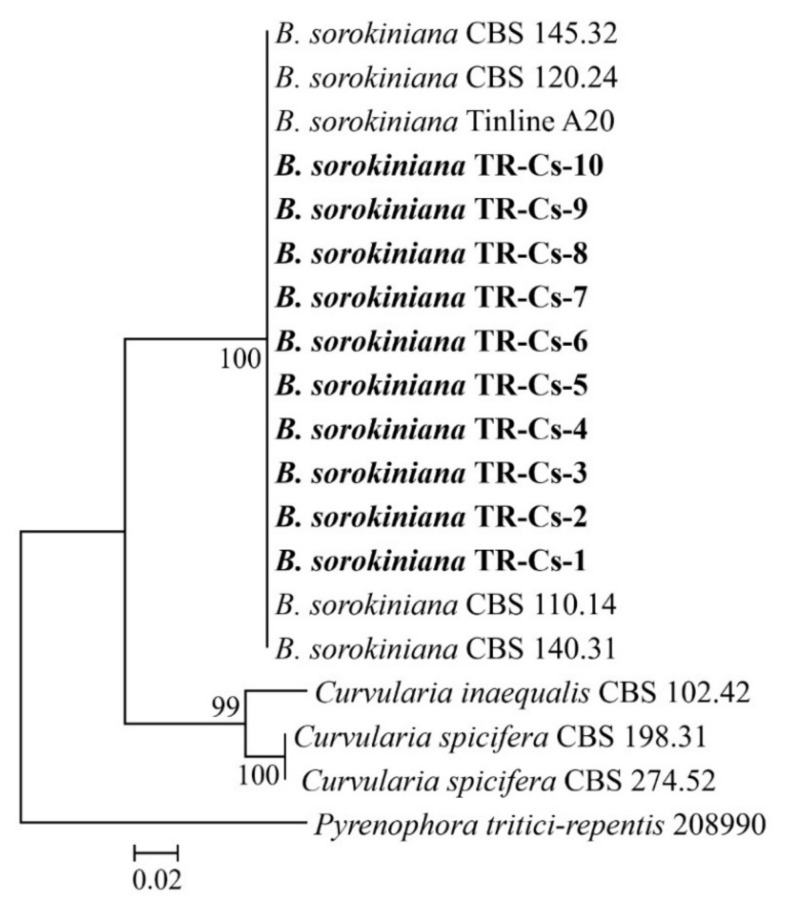
The Maximum Likelihood tree was generated using the ITS/*GAPDH* sequences of fungal isolates from this study (bold) and reference isolates derived from GenBank. The percentage of replicate trees in which the associated taxa clustered together in the bootstrap test (1000 replicates) is shown next to the branches.

*Bipolaris sorokiniana* occurred commonly in wheat fields located in Bolu province and was successfully isolated from all samples suggesting typical spot blotch symptoms (Figure 1). Based on morphological and cultural characteristics, the fungal identification was also confirmed molecularly by DNA sequencing of ITS and *GAPDH* gene region of ten isolates representing each different field. BLAST analysis of the 524 and 594 bp amplicons revealed 100% identity with ITS and *GAPDH* sequences of *B. sorokiniana* CBS 110.14, respectively. The resulting sequences were deposited in GenBank under accession numbers: MT271240-MT271249 for ITS and MW248907-MW248916 for *GAPDH*. The phylogenetic tree based on the ML method showed the isolates collected in this study, clustering with the reference isolates of *B. sorokiniana* derived from GenBank (Figure 2).

### 3.2. Aggressiveness of the Pathogen Isolates and Disease Reaction of Wheat 

Ten representative isolates from each field were subjected to the preliminary pathogenicity test on the susceptible cultivar Seri-82. All isolates caused typical spot blotch lesions on wheat leaves and disease index ranged from 3.5 to 5.5. Levene’s test indicated that there were statistically significant differences in the variance between all isolates (HSD = 0.856, *p* ≤ 0.05).

The most aggressive isolate TR-Cs-3 was selected to further evaluate disease reaction of 55 wheat cultivars, widely cultivated in Turkey. The results indicated that there were significant differences among the responses of wheat cultivars to the pathogen. The cultivars Anafarta and Koç-2015 were the most resistant with disease index of 1.63, while the cultivar Kırik was found to be the most susceptible cultivar with disease index of 7.97, followed by the cultivars Lancer and Doğu-88. Disease index of the other cultivars varied from 2.17 to 7.5.

### 3.3. Designing Species-Specific Primers

**Figure 3:**
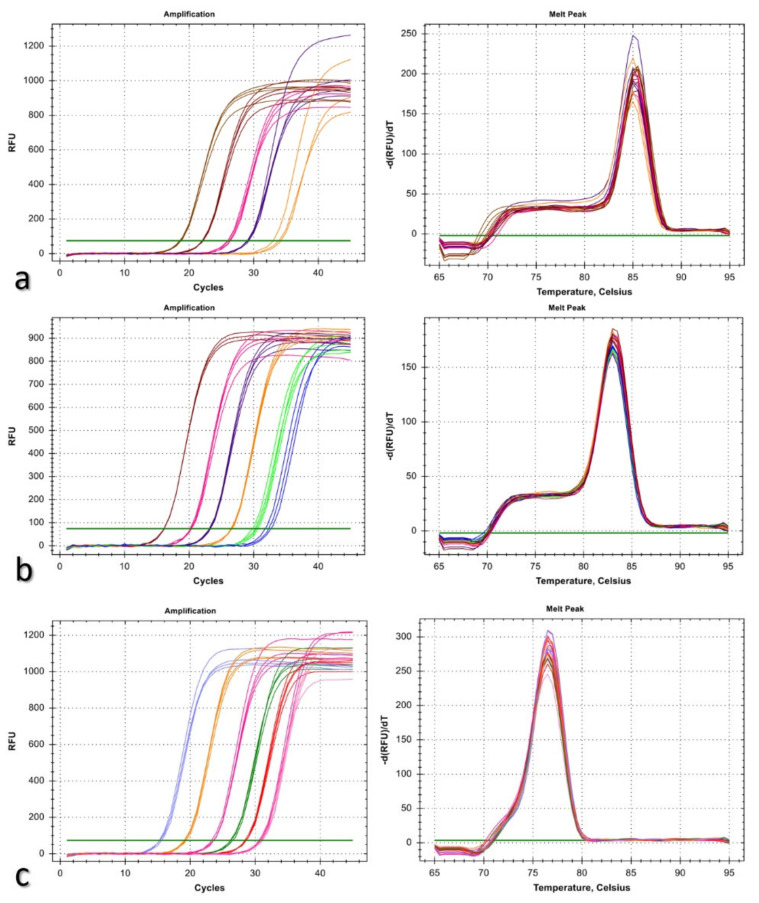
Real-time amplification profiles and melting curves for a 10-fold dilution series of *Bipolaris sorokiniana* genomic DNA, starting from 10 ng in 20 ng DNA of a healthy wheat plant using primers BsGAPDHF/BsGAPDHR (**a**), BsITSF/BsITSR (**b**), and BsSSUF/BsSSUR (**c**) designed in this study.

Conserved sequences of *ITS*, *GAPDH*, and *SSU* genes were selected as targets for determining the causal agent. Three primer sets, BsGAPDHF/BsGAPDHR, BsITSF/BsITSR, and BsSSUF/BsSSUR, were designed based on the sequence alignment of *B. sorokiniana* and the other fungi associated with wheat diseases (Table 2). The primer pairs BsGAPDHF/BsGAPDHR amplified a single product of 143 bp in size, while BsITSF/BsITSR and BsSSUF/BsSSUR primers produced a 108 and 130 bp amplicon from DNA samples of *B. sorokiniana* isolates, respectively. No cross-amplification occurred in DNA samples from the other fungi and plant tissues with these primer pairs. Detection limits of the primers were evaluated by using standard regression lines constructed from seven dilution series ranging from 10 ng to 0.01 pg (Figure 3). The detection sensitivity for BsGAPDHF/BsGAPDHR, BsITSF/BsITSR, and BsSSUF/BsSSUR primers was found as 1, 0.1, and 0.1 pg of pathogen DNA, while Cq values of the dilution series ranged from 18.54 to 33.69, 16.12 to 32.55, 15.36 to 31.12, respectively. The efficiency of qRT-PCR was 86.5% (GAPDH: *R*^2^ = 0.994, slope = −3.693, y-int = 22.212), 103.0% (ITS: *R*^2^ = 0.994, slope = −3.251, y-int = 19.933), and 110.2% (SSU: *R*^2^ = 0.980, slope = −3.100, y-int = 19.323). The melting curve analysis produced a single peak with dissociation temperature of approximately 85 °C, 83 °C, and 76.5 °C for BsGAPDHF/BsGAPDHR, BsITSF/BsITSR, and BsSSUF/BsSSUR primers, confirming the specificity of the PCR reaction, respectively (Figure 3).

### 3.4. Detection of Bipolaris sorokiniana Infection in Wheat Tissues

**Figure 4:**
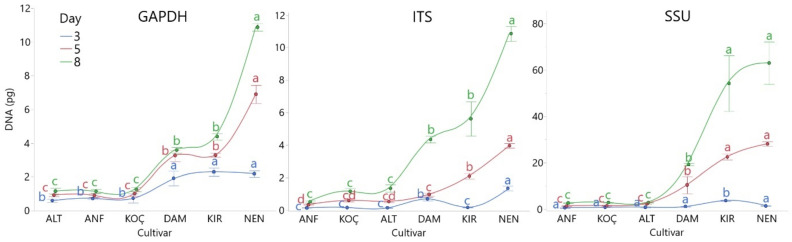
Quantification of *Bipolaris sorokiniana* infection in the resistant (ALT: Altay, ANF: Anafarta, KOÇ: Koç-2015) and susceptible (DAM: Damla, KIR: Kırik, NEN: Nenehatun) wheat cultivars using primers of BsGAPDHF/BsGAPDHR, BsITSF/BsITSR, and BsSSUF/BsSSUR on the third, fifth, and eighth days after inoculation. Each error bar is constructed using one standard error from the mean. Levels connected by the same letter on each line are not significantly different DNA amounts based on Tukey’s HSD.

The efficiency of primer pairs was also evaluated on the resistant (Altay, Koç-2015, and Anafarta) and susceptible (Kırik, Damla, and Nenehatun) wheat cultivars on the third, fifth, and eighth days after inoculation. All primer sets ensured the detection of fungal DNA in the early stages of pathogen infection. The obtained results indicated that there were significant differences among Cq values of the resistant and susceptible cultivars based on Tukey HSD test (*p* ≤ 0.01) (Figure 4). The mean DNA values detected in the qPCR assay with BsGAPDHF/BsGAPDHR, BsITSF/BsITSR, and BsSSUF/BsSSUR primers were 0.69, 0.13, and 0.74 in resistant cultivars on the third day after inoculation, while the mean values in susceptible cultivars were 2.34, 0.72, and 2.07, respectively. The amount of pathogen DNA increased gradually in the later days after inoculation. Additionally, the quantity of pathogen DNA in susceptible cultivars was higher than that in resistant cultivars for all primer sets on the fifth and eighth day after inoculation. No significant difference was observed in the amount of pathogen at different time points between resistant cultivars except for the fifth day with BsITSF/BsITSR primers, while the pathogen amounts in susceptible cultivars were variable for all days with all primers.

### 3.5. Differential Expression of Defense-Related Genes in Wheat Cultivars

**Figure 5:**
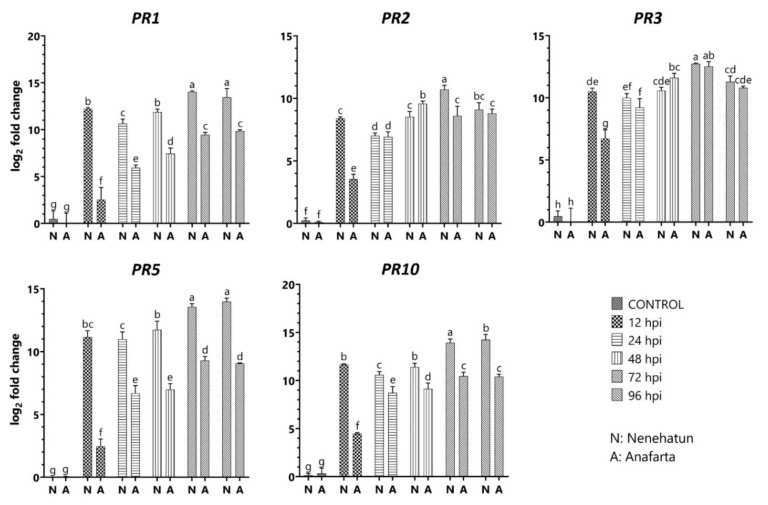
Expression profiles of the genes *PR1*, *PR2*, *PR3*, *PR5*, and *PR10* in susceptible (Nenehatun) and resistant (Anafarta) wheat cultivars at different time points post-inoculation of *Bipolaris sorokiniana*. Each column represents an average of three replicates, and error bars represent the standard error of means. Bars with different letters are significantly different from each other based on Student’s *t*-test analysis.

**Figure 6:**
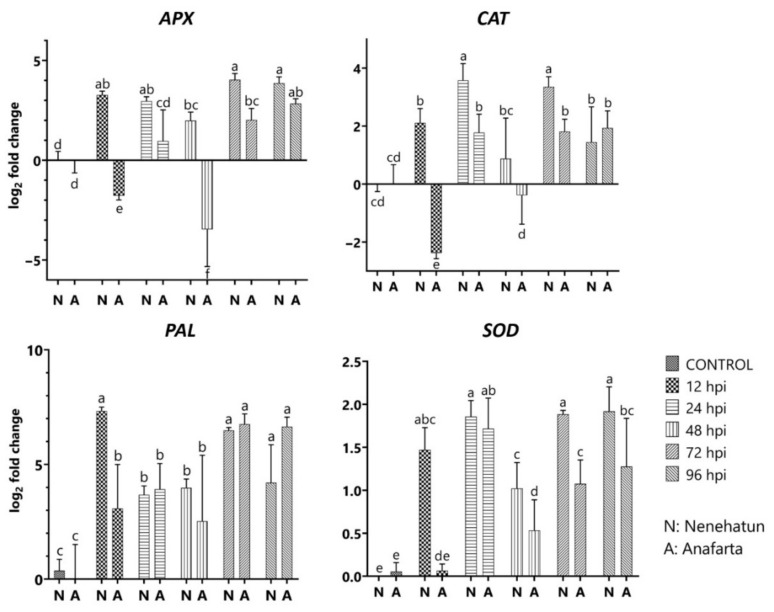
Expression profiles of the genes *APX*, *CAT*, *PAL*, and *SOD* in the susceptible (Nenehatun) and resistant (Anafarta) wheat cultivars at different time points post-inoculation of *Bipolaris sorokiniana*. Each column represents an average of three replicates, and error bars represent the standard error of means. Means indicated with different letters are significantly different from each other based on Student’s *t*-test analysis.

Expression levels of *PR1*, *PR2*, *PR3*, *PR5*, *PR10*, *PAL*, *CAT*, *SOD*, and *APX* genes were evaluated in the resistant cultivar ‘Anafarta’ and the susceptible cultivar ‘Nenehatun’ at different time points. The *PR1* gene was significantly upregulated in the defense response of both cultivars to the pathogen. The expression elevation was considerably higher in the susceptible cultivar Nenehatun compared to the resistant cultivar Anafarta (Figure 5 and Figure 6). The transcript level in Nenehatun elevated dramatically in the first 12 hpi and the maximum level was observed at 72 hpi with 14.03 of log_2_ fold change. The transcript level of *PR1* in the resistant cultivar was gradually upregulated from 12 to 72 hpi. The highest expression in the resistant cultivar at 96 hpi reached 9.85 log_2_ fold. The *PR2* level increased 3.55 and 8.4-log_2_ fold in Anafarta and Nenehatun at 12 hpi, respectively. The transcript level in both cultivars showed no significant differences at 24 and 96 hpi, while the expression enhancements at 12, 48, and 72 hpi were statistically different. The maximum activity in the resistant cultivar was observed at 48 hpi with 9.58 of log_2_ fold change. The highest level of *PR2* expression was reached in Nenehatun at 72 hpi. *PR3* transcript induced strongly in both cultivars but showed no significant difference between the cultivars except for 12 hpi. The highest expression of *PR3* gene in Nenehatun and Anafarta cultivars at 72 hpi reached 12.74 and 12.52-log_2_ fold, respectively. The transcript level of *PR5* was significantly upregulated in the susceptible cultivar than in the resistant cultivar at all inoculation point. *PR5* level showed a similar fluctuation ranging from 11.15 to 13.95 log_2_ fold in the susceptible cultivar for all time points, while the resistant cultivar, Anafarta, showed a weak elevation at 12 hpi and reached a peak of 8.53-log_2_ fold at 72 hpi. *PR10* transcript showed an expression profile similar to *PR5* regulation in both cultivars. The expression of *PR10* was significantly higher in Nenehatun than Anafarta at all time points. The highest expression of this gene in both cultivars at 96 hpi reached 16.25 and 10.4-log_2_ fold, respectively.

The transcript level of *PAL* attenuated rapidly and reached the maximum level in the susceptible cultivar at 12 hpi with 7.33 of log_2_ fold change, followed by a decrease until 48 hpi (Figure 6). The resistant cultivar showed a similar expression profile, ranging from 2.53 to 3.92-log_2_ fold until 48 hpi and strong induction of *PAL* gene with 6.76-log_2_ fold at 72 hpi. *SOD* activity in both cultivars remained below the baseline level at 12 and 48 hpi and the transcript level slightly increased at 24 hpi. Similarly, a slight induction was observed in the susceptible cultivar at 72 and 96 hpi, but not in the resistant cultivar. The expression enhancement of *CAT* activity in the susceptible cultivar continued until 24 hpi, followed by a decrease at 48 hpi, while *CAT* level increased again at 72 hpi about 3.36-fold. The transcript in the resistant cultivar was downregulated at 12 hpi. The highest expression of this gene in the resistant cultivar reached a peak of 1.94-log_2_ fold at 96 hpi but was not statistically different from that in the susceptible cultivar. *APX* activity induced 3.28-log_2_ fold in susceptible cultivar at 12 hpi, attenuated until 48 hpi, and reached the maximum level with 4.03-log_2_ fold at 72 hpi. The expression level in the resistant cultivar reduced −1.77 and −3.46-log_2_ fold at 12 and 48 hpi. The transcript was upregulated again at 72 hpi and reached 2.84-log_2_ fold at 96 hpi.

## 4. Discussion

Spot blotch, caused by *B. sorokiniana*, is a major disease of wheat and barley worldwide. The pathogen occurs widely in growing areas and causes important economic yield losses in cereals even to triticale that is adapted to harsh conditions [47]. In this study, the pathogen isolates from wheat fields in Bolu province were identified based on morphological characteristics and sequence analysis and evaluated for their aggressiveness. All isolates caused typical symptoms of spot blotch on inoculated wheat cultivar Seri-82 plants and indicated significant pathogenic variation, ranging from 3.3 to 5.5 of disease index. Similar results were observed in a previous study by Özer et al. [48], who reported that the disease severity of 96 isolates from the winter wheat-growing regions of Azerbaijan ranged from 1.58 to 3.60. Kang et al. [49] observed significant variation in the pathogenicity of 262 *B. sorokiniana* isolates from wheat samples in 97 locations of China. Similarly, variation in the aggressiveness of *B. sorokiniana* isolates was reported by several authors [8,50]. However, no considerable difference was found among the pathogenicity of *B. sorokiniana* isolates from Mexico and India [51,52]. In the current study, pathogenicity tests indicated that there were significant differences in the reactions of the 55 wheat cultivars to the TR-Cs-3isolate. The most resistant response was detected in the cultivar Anafarta and Koç-2015, while the cultivar Kırik showed the most susceptible reaction. Cultivation of these resistant cultivars in growing areas where the pathogen is common can be considered beneficial in improving the efficiency of disease management methods. Additionally, these may be potentially useful as genitor plants for breeding studies. Evaluating 625 wheat lines from a breeding program in India, Adlakha et al. [10] found 16 resistant lines. In China, resistant cultivars consisted of a low percentage among 21 wheat cultivars tested to the pathogen [49]. Testing 99 modern European winter wheat cultivars and breeding lines to four *B. sorokiniana* isolates revealed that the tested material had a low level of resistance [53]. These studies, which indicated the presence of limited resistance to the pathogen worldwide, increased the potential importance of resistance sources determined in this study.

*Bipolaris sorokiniana* exhibits a complex structure in wheat plants with other pathogens, such as *Pyrenophora tritici-repentis*, *Stagonospora nodorum*, *Blumeria graminis*, and *Septoria tritici*, which are a challenge to detect the main pathogen responsible for the disease in the early stages [54]. Classical diagnostic methods based on visual evaluation as well as microscopic and cultural methods provide limited benefit in the identification of fungal pathogens. Molecular methods have several advantages over the classical methods used to identify plant pathogens [14,16,55]. Various PCR-based methods have been developed to diagnose spot blotch disease of wheat [17,18,19]. In this study, a qPCR method was developed for detecting the pathogen in wheat tissue at the early stages of the infection process and for evaluating plant genotypes with different levels of resistance. Phylogenetically alignments of ITS, *GAPDH*, and SSU genes provided informative sites for separating *B. sorokiniana* and closely related fungi species from each other. Similarly, these conserved genes have widely been used in the taxonomic classification of *Bipolaris* species [15]. qPCR assay developed was highly sensitive and provided the determination of pathogen DNA up to 0.1 pg. The designed primer did not amplify DNA extracts from healthy plant tissue and other fungi species. Species-specific PCR developed by Matusinsky et al. [17] allowed detection of 0.001 ng of pathogen DNA, while a multiplex PCR (mPCR) system established for detecting wheat pathogens detected 100 pg for *B. sorokiniana* [56]. Real-time PCR assay described by Orina Aleksandra et al. [20] confirmed the presence of *B. sorokiniana* in 100% of the barley and oats samples and in 56% of wheat samples. The utility of the method was also demonstrated for confirming the presence of *B. sorokiniana* in plant samples at the early stages of infection and monitoring of resistant and susceptible plants as an optional method to visual scoring of disease severity. Our results showed a close relationship between the assessments of disease severity and the quantities of pathogen DNA in wheat cultivars. DNA quantities were observed at higher levels in susceptible genotypes than in resistant genotypes, suggesting this assay may possess an important potential for the discrimination of resistant and susceptible wheat genotypes. These results coincided with those of Bayraktar et al. [57], who observed a strong relationship between disease resistance and DNA quantification in resistant and susceptible chickpea cultivars to *A. rabiei* infection. Similarly, Daniëls et al. [58] designed a real-time PCR assay to evaluate host resistance to *Venturia inaequalis*. The results revealed a significant correlation between the resistance levels and DNA quantities of apple cultivars. Researchers reported that qPCR assay was the more robust and sensitive tool to assess resistance level of apple cultivars over the classical method. Leiminger et al. [59] evaluated the resistance of potato plants to *Alternaria solani* and *A. alternata* with classical and molecular methods. They found a significant correlation between the amount of pathogen DNA and the ratio of necrotic areas caused by *A. solani.*

The potential roles of *PR*s, *PAL*, *CAT*, *SOD*, and *APX* genes in defense response of wheat to *B. sorokiniana* were also investigated in both resistant and susceptible wheat cultivars. All the defense-related genes studied were upregulated in both cultivars except for the expression of *CAT* and *APX* genes at 12 and 48 hpi and showed considerable variation in their expression levels and timing. The upregulation was partially observed at higher levels in the susceptible cultivar than in the resistant cultivar. PR proteins, individually or in combination, have been widely reported to impair or uplift the level of defense response in plants to a wide range of pathogens [26]. *PR1* genes play a key role in disease formation as a result of host–pathogen interactions [29,60]. Our results showed the upregulation of *PR1* gene in defense response of both wheat cultivars, but more induced in the susceptible cultivar. Overexpression of *PR1* gene was detected in defense response of wheat to *Erysiphe graminis*, *ParaStagonospora nodorum*, and *Puccinia triticina* [33,61,62]. Soltanloo et al. [63] reported higher expression of *PR1* transcript in the resistant wheat genotypes compared to susceptible genotype upon infection with *F. graminearum*. Previous work carried out by Muhae-Ud-Din et al. [64] showed that the expression level of *PR1.1* transcript in defense response of wheat to *Tilletia controversa* was higher in the resistant cultivar than in the susceptible cultivar, while the expression of *PR1.2* was less expressed in the resistant cultivar than in the susceptible cultivar. *PR1* gene showed no or a slight induction in both resistant and susceptible wheat genotypes to yellow dwarf virus (YDV) aphid-transmitted and Hessian fly infestation, while a strong induction was observed to FHB-resistant cultivars Ning 7840 and KS24-1 and susceptible cultivar Len [65]. Additionally, the expression level of *PR1* gene in the susceptible cultivar Len was higher than that in the resistant cultivar Ning 7840, in agreement with the results of this study. *PR2* proteins (β-1,3-glucanases) that are directly involved in defense mechanisms by hydrolyzing the cell walls of fungal pathogens have been characterized from a wide range of species [27]. The upregulation of *PR2* gene was observed in wheat resistant to *P. triticina* and *F. graminearum* [66,67]. In this study, *PR2* gene contributed to resistance in both cultivars and reached the maximum level earlier in the resistant cultivar. Similarly, the increased expression of *PR1* and *PR2* was associated with enhanced resistance to rust pathogens in wheat [68]. However, Wu et al. [65] detected no significant change in the expression of *PR2* gene in both susceptible and resistant cultivars of wheat to *F. graminearum*. Our results also showed the potential involvement of *PR3*, *PR5*, and *PR10*, strongly inducing in both cultivars resistance pathways to *B. sorokiniana*. In wheat, *PR3*, which are chitinases, has been reported to be upregulated in response to fungal pathogens, *Puccinia* spp., *Blumeria graminis* [69], while overexpression of *PR5* gene referred to as thaumatin-like proteins provided to enhance wheat resistance to leaf rust [70]. The results obtained in the present study coincided with those of Zhang et al. [68], who reported that *PR1*, *PR2*, and *PR5* in wheat resistance to rust pathogens had a more significant role than *PR3* and *PR10* and the expression profiles of *PR* gene were different in response to different rust species or races of the same species. *PR10* was upregulated to *TaCAD12* transcript involving resistance response to sharp eyespot disease in wheat [28].

Expression of defense-related enzymes (*PAL*, *CAT*, *SOD*, and *APX*) is known to play a crucial role in the host resistance to pathogens. These enzymes are either directly or indirectly involved in plant defense pathways, such as the production of reactive oxygen species and secondary metabolites, and hypersensitive reactions. Our data presented the upregulation in gene expression levels of *PAL* and *SOD* in both cultivars to pathogen infection, but partially in the levels of *CAT* and *APX* in the resistant cultivar. Overexpression of *PAL* was detected in a resistant cultivar of soybean to *F. solani* f. sp. *glycine*, but not in the susceptible cultivar [71]. The silencing of *PAL* in wheat reduced aphid and nematode resistance [72], while PAL-RNAi in Brachypodium increased susceptibilities to the fungal pathogens *F. culmorum* and *Magnaporthe oryzae* [73]. However, the suppression of some *PAL* genes had no significant effect on plant resistance to *F. graminearum* [35]. Similar kinds of results that we observed were also reported by Christensen et al. [74], who indicated that overexpression of *TaGLP4* and *HvGLP4* with *SOD* activity enhanced resistance against *B. graminis* in wheat and barley, whereas transient silencing reduced basal resistance in both cereals. Our results are consistent with the results of Debona et al. [75], who detected the increased activities of *SOD*, *POX*, *APX*, and *GST* in both resistant and susceptible wheat cultivars infected with *Pyricularia oryzae* compared with noninoculated plants. Similarly, the antioxidant enzyme activities of the *POD*, *CAT*, and *APX* in resistant wheat genotypes were more efficient than susceptible genotypes to *Magnaporthe oryzae* [36]. Spanic et al. [34] observed differences in the antioxidant response of wheat varieties with different levels of resistance, namely an enhancement in *APX* and polyphenol oxidase (*PPO*) activity in FHB-resistant variety Vulkan in the early stages after infection, and a higher activity of *POD* and H_2_O_2_ in the moderately resistant variety Kraljica. Susceptible variety Golubica responded with enhanced *POD* activity to the pathogen.

In summary, we examined expression profiles of *PR*s, *PAL*, *CAT*, *SOD*, and *APX* genes in the resistance response of wheat to *B. sorokiniana*. Significant differences were observed depending on the timing and magnitude of these genes in wheat plants with different resistance levels. However, further investigation is necessary on other defense genes that play a role in plant defense mechanisms to gain more insight into the interaction of wheat pathogen.

## Figures and Tables

**Table 1:** List of wheat cultivars used in this study and their reactions to the most aggressive *Bipolaris sorokiniana* isolate TR-Cs-3.

Wheat Cultivar	Source	Wheat Type	Disease Severity *
Kırik	East Anatolian Agricultural Research Institute	Winter	7.97 ^a^
Lancer	East Anatolian Agricultural Research Institute	Winter	7.50 ^a^
Doğu-88	East Anatolian Agricultural Research Institute	Winter	6.27 ^b^
Nenehatun	East Anatolian Agricultural Research Institute	Winter	6.13 ^b^
Damla	Trakya Agricultural Research Institute	Winter	6.03 ^b^
Palandöken-97	East Anatolian Agricultural Research Institute	Winter	5.43 ^bc^
Karasu-90	East Anatolian Agricultural Research Institute	Winter	5.30 ^bd^
Ceyhan-99	Eastern Mediterranean Agricultural Research Institute	Spring	5.20 ^be^
Ayyıldız	East Anatolian Agricultural Research Institute	Winter	4.87 ^cf^
Es-26	Transitional Zone Agricultural Research Institute	Winter	4.60 ^cg^
Sarıbaşak	Eastern Mediterranean Agricultural Research Institute	Spring	4.60 ^cg^
Soyer-02	Transitional Zone Agricultural Research Institute	Winter	4.47 ^ch^
Müfitbey	Transitional Zone Agricultural Research Institute	Winter	4.47 ^ch^
Bezostaya-1	Transitional Zone Agricultural Research Institute	Winter	4.40 ^cı^
Seri-2013	Eastern Mediterranean Agricultural Research Institute	Spring	4.40 ^cı^
Nevzatbey	Black Sea Agricultural Research Institute	Winter	4.40 ^cı^
Adana-99	Eastern Mediterranean Agricultural Research Institute	Spring	4.37 ^cı^
Köprü	Trakya Agricultural Research Institute	Winter	4.33 ^cı^
Ekinoks	Eastern Mediterranean Agricultural Research Institute	Spring	4.27 ^dj^
İzgi-2001	Transitional Zone Agricultural Research Institute	Winter	4.26 ^dj^
Yüksel	Trakya Agricultural Research Institute	Winter	4.13 ^ek^
Bereket	Trakya Agricultural Research Institute	Winter	4.13 ^ek^
Gökkan	Eastern Mediterranean Agricultural Research Institute	Spring	4.03 ^fk^
Yunus	Transitional Zone Agricultural Research Institute	Winter	3.93 ^fl^
Alturna	East Anatolian Agricultural Research Institute	Winter	3.90 ^fm^
Gerek-79	Transitional Zone Agricultural Research Institute	Winter	3.90 ^fm^
Aldane	Trakya Agricultural Research Institute	Winter	3.87 ^fm^
Sönmez-2001	Transitional Zone Agricultural Research Institute	Winter	3.87 ^fm^
Candaş	Eastern Mediterranean Agricultural Research Institute	Spring	3.83 ^fn^
Alparslan	East Anatolian Agricultural Research Institute	Winter	3.83 ^fn^
Harmankaya-99	Transitional Zone Agricultural Research Institute	Winter	3.77 ^fn^
Sultan-95	Transitional Zone Agricultural Research Institute	Winter	3.77 ^fn^
Sakin	Black Sea Agricultural Research Institute	Winter	3.73 ^fn^
Kirve	Black Sea Agricultural Research Institute	Spring	3.60 ^go^
Nacibey	Transitional Zone Agricultural Research Institute	Winter	3.60 ^go^
Özcan	Black Sea Agricultural Research Institute	Winter	3.53 ^go^
Canik-2003	Black Sea Agricultural Research Institute	Winter	3.50 ^go^
Osmaniyem	Eastern Mediterranean Agricultural Research Institute	Spring	3.47 ^go^
Çetinel-2000	Transitional Zone Agricultural Research Institute	Winter	3.37 ^hp^
Daphan	East Anatolian Agricultural Research Institute	Winter	3.30 ^ıq^
Tekirdağ	Trakya Agricultural Research Institute	Winter	3.30 ^ıq^
Saban	Trakya Agricultural Research Institute	Winter	3.30 ^ıq^
Pehlivan	Trakya Agricultural Research Institute	Winter	3.13 ^jo^
Altındane	Black Sea Agricultural Research Institute	Spring	3.03 ^kq^
Mesut	Transitional Zone Agricultural Research Institute	Winter	2.87 ^lq^
Alpu-2001	Transitional Zone Agricultural Research Institute	Winter	2.87 ^lq^
Selimiye	Trakya Agricultural Research Institute	Winter	2.80 ^lq^
Altay	Transitional Zone Agricultural Research Institute	Winter	2.77 ^mr^
Yıldırım	East Anatolian Agricultural Research Institute	Winter	2.77 ^mr^
Altınbaşak	Eastern Mediterranean Agricultural Research Institute	Spring	2.70 ^mr^
Abide	Trakya Agricultural Research Institute	Winter	2.53 ^or^
Gelibolu	Trakya Agricultural Research Institute	Winter	2.27 ^pr^
Yakamoz	Eastern Mediterranean Agricultural Research Institute	Spring	2.17 ^qr^
Koç-2015	Bati Akdeniz Agricultural Research Institute	Spring	1.63 ^r^
Anafarta	Trakya Agricultural Research Institute	Winter	1.63 ^r^

* The values with the same letters are not significantly different from each other based on the HSD (*p* = 0.05).

**Table 2:** Sequences of primers used for the detection of *Bipolaris sorokiniana* and the evaluation of the expression level of defense-related genes in wheat.

Target Genes	Primer Names	Sequence (5′–3′)	References
ITS	BsITSF	TTCTGGGAGACTCGCCTTA	
	BsITSR	GTCTTGATGGATTACCGTCCTT	
*GAPDH*	BS_F01	CCATTCACGCATATTAAAGCTG	in this study
	BS_R01	CTCTGGTGAAAGGTTCTGGATT	
SSU	BsSSUF	GCGAAGGCAAACCTCTATGTA	
	BsSSUR	CGTCCCTCAACGTCAGTTATAG	
*β-tubulin*	β-tubulin_F	GCCATGTTCAGGAGGAAGG	[45]
	β-tubulin_R	CTCGGTGAACTCCATCTCGT	
*PR1*	TaPR1_F	GAGAATGCAGACGCCCAAGC	
	TaPR1_R	CTGGAGCTTGCAGTCGTTGATC	
*PR2*	TaPR2_F	AGGATGTTGCTTCCATGTTTGCCG	
	TaPR2_R	AAGTAGATGCGCATGCCGTTGATG	
*PR3*	TaPR3_F	TACTGCTTCAAGGACCAGATAGAC	
	TaPR3_R	CACCAGGTTCGGGTTGTTTA	
*PR5*	TaPR5_F	CAAGCAGTGGTATCAACGCAGAG	
	TaPR5_R	GTGAAGCCACAGTTGTTCTTGATGTT	
*PR10*	TaPR10_F	TTAAACCAGCACGAGAAACATCAG	
	TaPR10_R	ATCCTCCCTCGATTATTCTCACG	
*Phenylalanine ammonia-lyase*	TaPAL_F	CGTCAAGAGCTGTGTGAAGATGG	[46]
	TaPAL_R	GGTAGTTGGAGCTGCAAGGGTC	
*Catalase*	TaCAT_F	TGCCTGTGTTTTTTATCCGAGA	
	TaCAT_R	CTGCTGATTAAGGTGTAGGTGTTGA	
*Superoxide dismutase*	TaSOD_F	CGATAGCCAGATTCCTTTGACT	
	TaSOD_R	GAAACCAGCGACCTACAACG	
*Ascorbate-peroxidase*	TaAPX_F	GGTTTGAGTGACCAGGACATTG	
	TaAPX_R	GCATCCTCATCCGCAGCAT	

## Data Availability

All relevant data generated or analyzed during this study are included in this manuscript.
